# The safety of fertility and ipsilateral ovary procedures for borderline ovarian tumors

**DOI:** 10.18632/oncotarget.23021

**Published:** 2017-12-06

**Authors:** Tong Lou, Fang Yuan, Ying Feng, Shuzhen Wang, Huimin Bai, Zhenyu Zhang

**Affiliations:** ^1^ Department of Obstetrics and Gynecology, Beijing Chao-Yang Hospital, Capital Medical University, Beijing, China; ^2^ Department of Obstetrics and Gynecology, the affiliated hospital of Qingdao University, Qingdao, China

**Keywords:** borderline ovarian tumor, BOT, recurrence, survival, treatment

## Abstract

**Objective:**

To explore the optimal treatment options for women with borderline ovarian tumors (BOTs).

**Materials and Methods:**

The medical records of consecutive patients with BOTs in two academic institutions were retrospectively collected. The pertinent data, including clinicopathological characteristics and, treatment and prognostic information were evaluated.

**Results:**

A total of 281 cases of BOTs were included in this analysis. For the entire series, the 5- year disease-free survival (DFS) and overall survival (OS) rates were 91.8% and 98.5%, respectively. In the multivariate analysis, reservation of the ipsilateral ovary (HR: 0.104 [95% CI, 0.036–0.304], *p* = 0.000) and FIGO stage II–III (HR: 6.811 [95% CI, 2.700–17.181], *p* = 0.000) were the independent risk factors for recurrence. Ovarian surface involvement (HR: 64.996 [95% CI, 4.054–1041.941], *p* = 0.003) was the only independent prognostic factor for OS. Lymphadenectomy and adjunct chemotherapy had no significant impact on patients’ recurrence and survival (recurrence: *p* = 0.332 and 0.290, respectively, survival: *p* = 0.896 and 0.216, respectively).

**Conclusions:**

Fertility-sparing surgery with healthy ovarian preservation seems safe and feasible for young women who prefer fertility-sparing treatment. Ovarian cystectomy to conserve the affected ovary/ovaries without ovarian surface involvement may be cautiously performed under fully informed consent for young women with bilateral BOTs who strongly prefer fertility-sparing treatment and have no evidence of infertility. However, long-term follow-up is necessary due to the relapse susceptibility of the ovary.

## INTRODUCTION

Borderline ovarian tumors(BOTs) were first reported by Taylor in 1929, and have been considered to be distinct types of ovarian tumors by the World Health Organization since the 1970s [1]. BOTs account for 10%-15% of all ovarian tumors [2]. In recent decades, the incidence of BOTs has been rising [3], possibly due to improved diagnostic accuracy, and the use of fertility drugs and contraceptives [4, 5]. BOTs are non-invasive tumors displaying epithelial proliferation, cytological atypia and low malignancy [6], consisting of serous, mucinous, endometrioid, clear cell and Brenner subtypes, among others. BOTs are common in women of child-bearing age [7–9], and are usually limited to the ovary in 80% of cases [10, 11]. The prognosis of BOTs was much more favorable than that of malignant ovarian tumors. The 5-year survival rates for women with stage I BOTs were as high as 95%-97%, and these rates were even as high as 65–87% for those with more advanced disease (stages II–III) [12].

The current recommended treatment for this relative inert tumor is a hysterectomy and bilateral adnexectomy, which frequently leads to a clinical dilemma in treating young women who have not yet given birth. Given these circumstances, less radical surgeries, such as fertility- or ovary-sparing procedures, may be preferred and are still safe treatment modalities in selected patients. In addition, the prognostic role of lymphadenectomy for complete surgical staging is also debated [13], since the reported risk of lymphatic metastasis is 0.3–13.7% [14–16]. Due to the slow growth rate of tumor cells, the necessity of adjuvant chemotherapy has also been controversial.

In this study, we tried to investigate the clinicopathological characteristics and clinical outcomes of patients with BOTs, with the goal of identifying a subset of patients possibly suited for less radical surgery, and/or lymphadenectomy and chemotherapy omission.

## RESULTS

During the study period, 308 consecutive patients were diagnosed with and treated for BOTs at the two hospitals. Fifteen (4.9%) patients suffering from a primary malignant tumor in another part of the body (12 cases) or other types of ovarian malignancies cell (3 cases) were excluded. Twelve (3.9%) patients lost to follow-up within one month after the initial surgery were also excluded. Thus, a total of 281 patients met the inclusion criteria for further analysis. The demographic and clinicopathologic characteristics are presented in Table 1 and Table 2, respectively. The median age at diagnosis was 38 years (range: 13–86 years). Most (72.2%) of patients were pre-menopausal. Seventy-four patients (26.3%) were nulligravida, including 12 patients (4.3%) with infertility. The most common presentation was abdominal pain (28.5%), followed by abdominal distension (12.1%), but more than half (54.4%) of the patients were asymptomatic. An elevated level of preoperative CA125 was identified in 122 (43.4%) patients.

**Table 1:** Demographic and surgical characteristics of patients with borderline ovarian tumors

Parameter	Number of patient	Percent (%)
Age at diagnosis, (Mean; range)	38 (13–86)	
Menopause status		
Pre-menopause	203	72.2
Post-menopause	78	27.8
History of infertility		
Yes	12	4.3
No	269	95.7
Complaint at admission		
Abdominal pain	80	28.5
Abdominal distension	34	12.1
Abnormal vaginal bleeding	9	3.2
Urinary frequency	5	1.8
Asymptomatic	153	54.4
Pre-treatment CA125 level (U/ml, median, range)	34.9 (1.54–9960)	
< 35 U/ml	117	41.6
≥ 35 U/ml	122	43.4
Not performed	42	14.9
Surgery type (*n*,%)		
Fertility-sparing surgery	138	49.1
Radical surgery	143	50.9
Involvement ovary (*n*, %)		
Reserved	70	24.9
Excision	211	75.1
Completely Staging surgery (*n*, %)		
None	163	58.0
Yes	118	42.0
Received postoperative chemotherapy	35	12.5
Recurrence (*n*, %)	20	7.1
Recurrence site		
Ovary/Ovaries	15	75.0
abdomen or pelvis	7	46.7
Lymph node	2	10.0
Liver	1	5.0
Treatment after recurrence (*n*,%)		
Surgery	16	80.0
Chemotherapy	9	45.0
Not known	2	10.0
Recurrence interval (months)	29.0 (2–133)	
Disease-free survival (*n*, range in months)	41.0 (2–190)	
Overall survival (*n*, range in months)	43.0 (5–237)	

CA125 = cancer antigen 125.

**Table 2:** Pathological characteristics of patients with borderline ovarian tumors

Parameter	Number of patient	Percent (%)
Frozen section diagnosis		
benign	60	21.4
borderline	197	70.1
malignant	8	2.8
Not done	16	5.7
Tumor size (median, range in cm)	10 (2–50)	
≤ 8 cm	109	38.8
> 8 cm	158	56.2
Histology		
Serous	159	56.6
Mucinous	98	34.9
Seromucinous	12	4.3
Endometrioid	5	1.8
clear cell	4	1.4
Brenner	3	1.1
FIGO stage (*n*, %)		
IA	195	69.4
IB	8	2.8
IC	52	18.5
II	9	3.2
III	17	6.0
Ascites cytology positive	18	6.4
Micro interstitial infiltrates		
Yes	17	6.0
No	264	94.0
Peritoneal implantation		
No	262	93.2
Yes	19	6.80
Non-invasion	7	36.8
Invasion	12	63.2
Primary lesion side		
Unilateral	252	89.7
Left	96	38.1
Right	156	61.9
Bilateral	29	10.3
LNM		
Yes	8	2.8
No	273	97.2
Ovary surface involvement		
Yes	3	1.1
No	278	98.9

USO = Unilateral salpingo-oophorectomy; LNM = Lymph node metastasis

A rapid-freezing section histological examination was performed in 265 (94.3%) patients, with a sensitivity of 74.3% for the diagnosis of BOT. Total hysterectomy and bilateral salpingo-oophorectomy was performed in 143 (50.9%) patients. The remaining 138 (49.1%) women received fertility-sparing surgery. Ovarian cystectomy to reserve the ipsilateral ovary/ovaries was performed in 70 of these patients. Lymphadenectomy for complete staging was performed in 118 (42%) patients. No macroscopic residual tumor was left within the abdominopelvic cavity after the initial surgery.

The median tumor size was 10 (range: 2–50) cm. Tumors confined within the unilateral ovary were identified in 252 patients (89.7%), in most patients (61.9%), tumors were located on the right ovary. The spontaneous rupture (9 cases) or surgical spill (40 cases) of tumors occurred in 40 (14.2%) patients. The most common histological subtype was the serous tumor (56.6%), followed by the mucinous (34.9%) tumor. The other subtypes were quite rare (8.6%). Microscopic interstitial infiltration was identified in 17 (6.0%) patients, and tumor cells on the surface of the ovary were found in 3 (1.1%) patients. Peritoneal implantation was identified in 19 (6.8%) patients, in the omentum (10 patients), pelvic peritoneum (15 patients), abdominal wall (2 patients) and appendix (1patient). Twelve cases involved invasive implantation. Lymph node metastasis (LNM) was detected in 8 (2.8%) patients and no patients had lymphadenectasis during surgery. None of the patients had ascites in our study. Washings were collected prior to the operations in all the patients, and atypical cells were identified in 18 patients (6.4%). The FIGO staging was distributed as follows: 255 (90.7%) cases were stage I, 9 (3.2%) cases were stage II, 17 (6.0%) cases were stage III, and no one was stage IV. More than half (69.4%) of patients had stage IA disease.

Thirty-five patients (12.5%) received adjuvant chemotherapy, due to LNM (6 cases), invasive implants (4 cases), positive pelvic washings (5 cases), rupture of ovarian cysts (15 cases), ovarian surface involvement (1 case), and microscopic interstitial infiltrates (4 cases). The cytotoxic agents were well-tolerated.

The median follow-up for this analysis was 43.0 (range: 5–237) months. At the last contact, 20 patients (7.1%) experienced relapse. The recurrent interval was 29.0 (range: 2–133) months. The sites of recurrent disease included the ovary (15 patients), abdomen or pelvis peritoneum (7 patients), lymph nodes (2 patients), and liver (1 patient). Secondary surgery was administered in 16 (80%) patients, and optimal cytoreductive surgery (CRS) was achieved in 11 of them. Five young patients received an ipsilateral ovarian reservation procedure again under fully informed consent. Salvage chemotherapy was performed in 9 patients, and 4 (44.4%) of these patients showed an objective partial response. Two (10%) remaining patients could not afford further aggressive treatment due to multiple sites of metastasis and very poor physical condition, and one of these patients died of the disease eventually. Over time, 11 patients achieved a tumor control again, but 4 (1.4%) patients died of the disease. The remaining 5 (1.80%) patients were alive but still had tumors. Thus, a total of 272 (96.8%) patients survived without any evidence of disease at the time of the last visit. The clinicopathological characteristics of the 4 patients who died of the disease are shown in Table 4.

**Figure 1:**
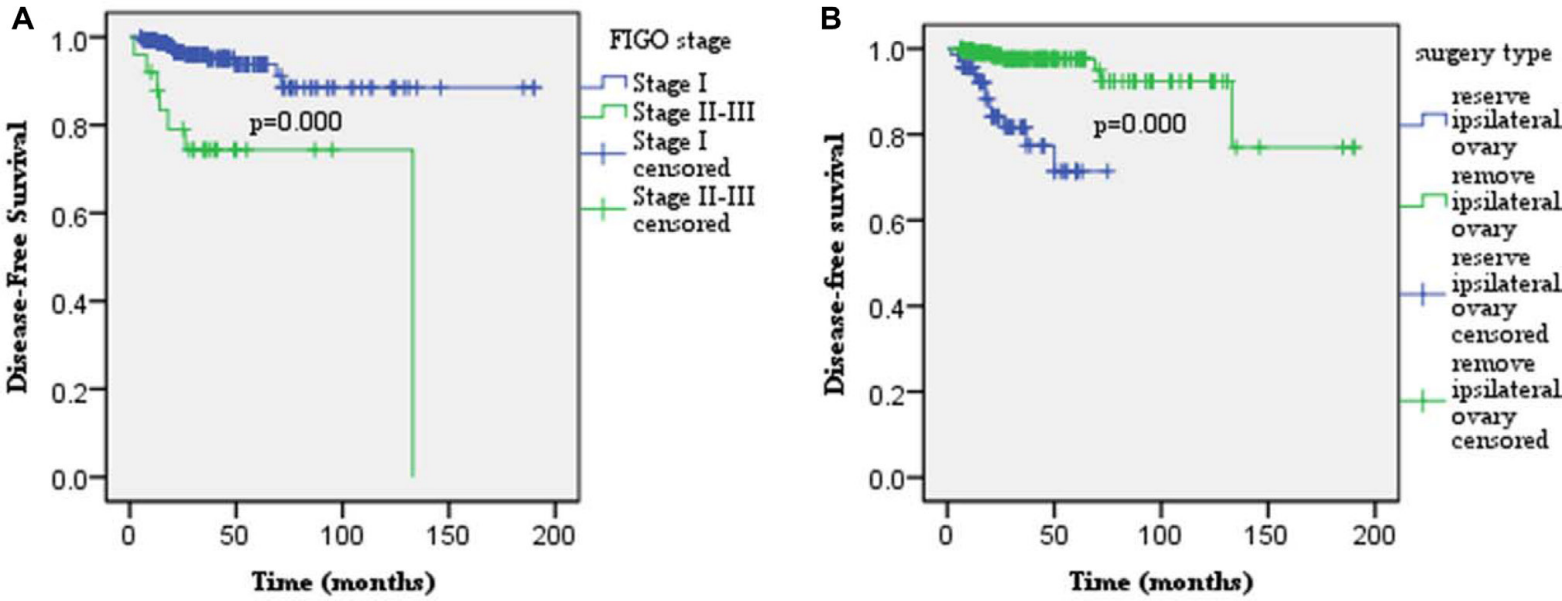
Multivariate analysis revealed that FIGO stage II–III (**A**) and ipsilateral ovary reservation (**B**) were identified as the independent adverse factors for recurrence (*p* = 0.000, and 0.000, respectively).

**Figure 2:**
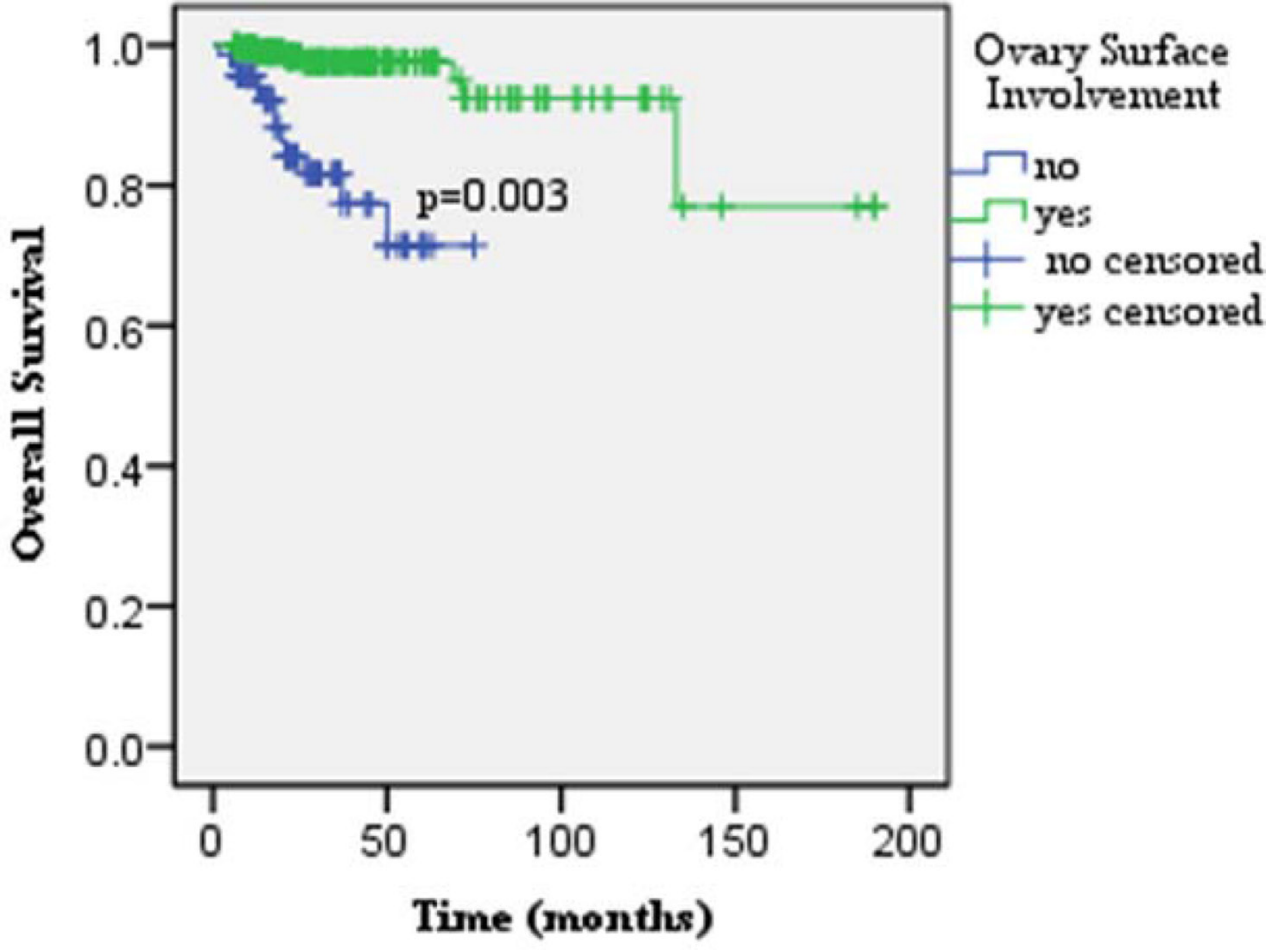
Ovarian surface involvement was identified as the only independent risk factor for patients’ survival in the multivariate analysis (*p* = 0.003)

For the entire series, the 5- year DFS and OS rates were 91.8% and 98.5%, respectively. Older age, fertility-sparing surgery, ipsilateral ovary-sparing surgery, FIGO II-III disease, peritoneal implantation, ovarian surface involvement, and LNM significantly decreased patients’ DFS in the univariate analysis (*p* < 0.01, < 0.01, < 0.01, < 0.01, = 0.001, = 0.034, and = 0.013, respectively; Table 3). Ovarian surface involvement, completely staging surgery and ovarian tumor rupture were the significant predictors of OS (*p* < 0.01, = 0.008, and = 0.034, respectively). Multivariate analysis, revealed that FIGO stage II–III and ipsilateral ovarian reservation were identified as the independent risk factors for recurrence (*p* = 0.000, and 0.000, respectively, Figure 1). For patients with stage I disease who had received ipsilateral ovary resection, the 5-year DFS was as high as 98.7%, compared with 67.9% for patients with stage II-III diseases or positive ovarian surface involvement, receiving ipsilateral ovary-sparing surgery. Ovarian surface involvement was independently associated with patients’ survival in the multivariate analysis (*p* = 0.003; Figure 2). The 5-year OS rate in the ovarian surface involvement subgroup was 0.0% vs. 99.3% in the negative ovarian surface subgroup. Lymphadenectomy and adjunct chemotherapy had no significant impact on patients’ recurrence and survival (recurrence: *p* = 0.896 and 0.216, respectively; survival: *p* = 0.332 and 0.290, respectively).

**Table 3:** The survival analysis of the effect of clinical pathological characteristics on prognosis in borderline ovarian tumors

Parameter	Relapse		*p* value^a^	*p* value^b^	DOD		*p* value^c^	*p* value^d^
	+	−			+	−		
Age								
< 40	16	130	0.004	0.558	3	143	0.533	-
≥ 40	4	131			1	134		
Menopause								
Yes	2	76	0.084	-	1	77	0.887	-
No	18	185			3	200		
Infertility								
Yes	1	11	0.648	-	0	12	0.792	-
No	19	250			4	265		
Pre-treatment CA125								
< 35 IU/ml	5	112	0.461	-	3	114	0.115	-
≥ 35 IU/ml	7	115			1	121		
Surgery type								
Fertility-sparing surgery	17	121	0.000	0.315	2	136	0.884	-
Radical surgery	3	140			2	141		
Involved ovary								
Preserved	13	57	0.000	0.000	0	70	0.416	-
Removed	7	204			4	207		
Staging surgery								
No	13	150	0.332	-	2	161	0.008	0.893
Yes	7	111			2	116		
Primary lesion side								
Unilateral	17	235	0.412	-	4	248	0.467	-
Bilateral	3	26			0	29		
Tumor size (cm)								
≤ 8	3	106	0.439	-	1	108	0.667	-
> 8	8	150			3	155		
Ovarian tumor ruptured								
None	17	215	0.733	-	2	230	0.034	0.246
Spontaneous rupture	1	8			1	8		
Intraoperative rupture	2	38			1	39		
Lymphadenectomy								
Remove or biopsy	7	111	0.332	-	2	116	0.896	-
No	13	150			2	161		
FIGO staging system								
I	12	243	0.000	0.000	3	252	0.293	-
II–III	8	18			1	25		
Pelvic rinses or celiac cytology								
Positive	1	17	0.694	-	1	17	0.255	-
Negative	19	244			3	260		
Micro interstitial infiltrates								
Yes	2	15	0.288	-	0	17	0.604	-
No	18	246			4	260		
Peritoneal implantation								
No	15	247	0.001	0.548	4	258	0.580	-
Yes	5	14			0	19		
Non-invasion	2	5	0.549	-	0	7	-	-
Invasion	3	9			0	12		
Surface involvement								
Yes	1	2	0.034	0.071	1	2	0.000	0.003
No	19	259			3	275		
LNM								
Yes	2	6	0.013	0.640	0	8	0.958	-
No	18	255			4	269		
Chemotherapy								
Yes	6	14	0.290	-	2	18	0.216	-
No	14	247			2	259		

DOD = death due to disease; cm = centimeter; LNM = lymph node metastasis.

^a^: single factor analysis of clinical pathological characteristics and disease-free survival.

^b^: multifactor analysis of clinical pathological characteristics and disease-free survival.

^c^: single factor analysis of clinical pathological characteristics and overall survival.

^d^: multifactor analysis of clinical pathological characteristics and overall survival.

**Table 4:** Four patients with recurrence borderline ovarian tumor that developed death

Parameter	Case 1	Case 2	Case 3	Case 4
Age	58	36	17	38
Menopause status	Post-menopause	Pre-menopause	Pre-menopause	Pre-menopause
Tumor size(cm)	25	8	12	4
Pre-treatment CA125(IU/ml)	14.11	18.81	217.5	14.14
Histology	mucinous	serous	mucinous	mucinous
FIGO stage	IA	IA	IIA	IC3
Primary surgery type	radical	conservative	conservative	radical
Primary surgery	TH-BSO + Om + PC	PC + USO and contralateral ovarian biopsy	USO + PC	TH-BSO + Om + PC + App +P LND
Laterality	unilateral	unilateral	unilateral	unilateral
Appendectomy	No	Yes	Yes	Yes
Lymphadenectomy	No	Yes	No	Yes
LNM	-	No	-	No
Abdominal dropsy cytology	Negative	Negative	Negative	Positive
Micro interstitial infiltrates	No	No	No	No
Peritoneal implantation	No	No	Yes	No
Surface involvement	No	No	Yes	No
Chemotherapy	No	No	Yes	Yes
Treatment after recurrence	secondary CRS and chemotherapy	chemotherapy	secondary CRS and chemotherapy	No treatment
Recurrence intervel (months)	21	69	26	72
Overall survival (months)	33	74	39	75

TH: total hysterectomy; BSO: bilateral salpingo-oophorectomy; USO: unilateral salpingo-oophorectomy; App: appendectomy; Om: omentectomy; PC: peritoneal cytology; PLND: pelvic lymph node dissection; LNM= Lymph node metastasis; CRS= cytoreductive surgery

## DISCUSSION

BOTs were associated with favorable prognosis. Based on our data, 5-year DFS and OS were as high as 91.8% and 98.5%, respectively, and the recurrence rate was only 7.1%, which was between the rates of 5%-16.8% reported in the literature [17–21]. In this study, FIGO stage II-III and ipsilateral ovarian reservation were identified as the independent risk factors for recurrence. Ovarian surface involvement was the only independent risk factor for OS. Our data showed that the 5-year OS rate in the negative ovarian surface involvement subgroup was as high as 99.3%, compared to 0.0% in the positive ovarian surface subgroup.

Total abdominal hysterectomy and bilateral salpingo-oophorectomy (BSO) have been regarded as the standard treatment for BOT [22–24]. In this analysis, the most common recurrent site is the ovary (75%), consistent with findings from previous studies [25, 26]. In addition, the univariate analysis showed that a fertility-sparing procedure was a significant risk factor for patient relapse. Thus, radical surgery and complete resection of the macroscopic lesion still should be considered as options for patients with adverse prognostic factors, such as an advanced stage (FIGO stage II–IV) and ovarian surface involvement [18, 20].

BOTs were very common in young women [18, 19, 21, 27]. In this study, the median age at diagnosis was 38 years, and approximately one-fourth of patients were nulligravida. The prognostic impact of fertility-sparing on BOT patients is still controversial. Several studies demonstrated that fertility-sparing surgery was an independent adverse predictor for recurrence [28, 29]. However, other researchers hold the opposite view [20, 21]. The explanation for this “paradox” may be that ovarian cystectomy to preserve the ipsilateral ovary (ies) was not stratified from fertility-sparing procedures in the analysis in these studies. In this study, fertility-sparing surgeries were associated with a high risk of recurrence in the univariate analysis, yet were excluded from the model in the multivariate analysis, as a confounding factor. Ovarian cystectomy for preserving the ipsilateral ovary/ovaries was identified as one of the independent adverse factors for recurrence. Thus, fertility-sparing surgery with healthy ovarian reservation was safe and feasible for young women who wanted to be able to conceive. However, long-term follow-up was necessary due to the relapse susceptibility of the ovary. For patients with recurrent disease, secondary CRS with a castration procedure was recommended.

Bilateral ovarian tumors are relatively rare but not negligible [30], and they accounted for 10%, of the cases in this analysis. The safety of ipsilateral ovarian reservation for BOTs has been seldom evaluated in the literature. Ovarian cystectomy to spare the ipsilateral ovary/ovaries improved the risk of recurrence [2, 15, 31, 32], consistent with our findings. However, this procedure had no significant impact on OS in patients with BOTs. Consequently, ovarian cystectomy may be cautiously performed under fully informed consent for young women with bilateral BOTs without ovarian surface involvement under fully informed consent who strongly prefer fertility-sparing treatment. Close long-term follow-up was mandatory given the high risk for recurrence. In addition, the use of extreme caution when performing cystectomy remains critical to avoid intraoperative tumor rupture, although the spillage of tumor cells did not have a significant adverse impact on survival in women with BOTs. Given the currently limited data, the safety and feasibility of this management necessitates further evaluation.

The treatment role of lymphadenectomy for BOTs was also controversial. LNM was quite rare in BOTs. The rate of LNM was 2.8% in this study, which was between the reported rates (0.3–13.7%) in the literature [14–16]. In contrast, Benedicte et al. [33] found that this parameter in the subgroup of advanced stage was approximately as high as 28% through consulting the US Surveillance, Epidemiology, and End Results (SEER) database. This analysis validated the findings of previous studies [33, 34] that lymphadenectomy made no difference in the clinical outcomes of patients with BOTs. However, node involvement was associated with a significantly adverse progression-free survival (PFS) [35, 36]. In our study, 2 of 20 (10%) patients developed recurrence on lymph nodes, suggesting the moderate relapse risk of lymph nodes. Thus, lymphadenectomy should still be recommended for patients with advanced stage disease and/or with ovarian surface involvement with the purpose of either providing prognostic information or guiding postoperative treatment.

For BOTs, consensus has also not been reached on the indications and roles of adjuvant chemotherapy. This treatment method has not been identified in this study or in previous studies as a significant favorable factor for the prognosis of patients with BOTs [15, 26]. Trope et al. [37] demonstrated that adjuvant therapy showed no prognostic benefit but had serious toxicity. This absence of significance might be ascribed to the fact that adjuvant therapies had a greater likelihood of being performed for patients with adverse factors for relapse and survival [38, 39]. In addition, in this analysis, salvage chemotherapy was performed in 9 patients, and 4 (44.4%) of these patients showed an objective partial response. Thus, adjuvant treatment still should be reserved for patients with ovarian surface involvement and /or advanced-stage diseases.

Despite its retrospective nature, a strength of this study is the relative completeness of the pathological reports and follow-up information. In addition, this analysis spans the past 16 years, reflecting the latest treatment strategies for this disease. These strengths enabled us to perform robust analyses to evaluate the impact of different clinicopathological features of BOTs on patients’ survival.

In conclusion, ovarian surface involvement was the only independent risk factor for the survival of patients with BOTs. Radical surgery and complete resection of the macroscopic lesion should still be regarded as the standard treatment. Fertility-sparing surgery with healthy ovarian reservation seems safe and feasible for young women who desire fertility-sparing treatments. Ovarian cystectomy to conserve the affected ovary/ovaries without ovarian surface involvement may be cautiously performed under fully informed consent for those with bilateral BOTs who strongly prefer fertility-sparing treatment and have no evidence of infertility. However, long-term follow-up is necessary due to the relapse susceptibility of the ovary.

## MATERIALS AND METHODS

The medical records of all consecutive patients diagnosed and treated for BOTs from January 2001 to December 2016 at two cancer referral centers, including Beijing Chao-Yang Hospital, Capital Medical University, and the affiliated hospital of Qingdao University, were retrospectively reviewed. Patients suffering from a primary malignant tumor in another part of the body or other malignant ovarian cell types were excluded. Patients without complete surgery and pathology reports or who were lost to follow-up within one month after the initial surgery were also excluded from this study. Patient information, including demographic and pathological characteristics, and disease status at the last contact, was collected and evaluated.

Serum CA-125 served as both a pre- and postoperative tumor marker, and rising levels were defined as a progressive increase in three consecutive serum antigen values above 35 U/ml. The major initial surgical procedure was complete staging surgery or cytoreductive surgery (CRS), which consisted of total hysterectomy, bilateral salpingo-oophorectomy, and omentum, and peritoneum multiple-site biopsies with lymph node sampling or dissection. Optimal CRS was defined such that the largest diameter of residual lesions within the abdominopelvic cavity was no more than 1cm. Ascites or washings were routinely collected prior to the operation, and cytological data were evaluated. Fertility-sparing surgery was performed under fully informed consent for young women who desired fertility preservation. When the tumor was confined within the unilateral ovary, the contralateral ovary was preserved in young women. For those with bilateral tumors, the clinical decision regarding uni- or bilateral ovarian cystectomy or bilateral salpingo-oophorectomy was generally made based on the patients’ age, their informed consent, the institutional practice at the time, and the doctor’s’ advice.

Two independent pathologists with extensive gynecological pathology backgrounds reviewed all pathological slides; these two pathologists were blinded to the patients’ outcomes. Tumor size was defined as the longest diameter of the tumor during the operation. A BOT was defined by the following characteristics [33]: (1) epithelial cells were typical, and a stratified lining was present; (2) nuclear atypia was present, and the degree of nuclear division was between benign and malignant; and (3) above all, frank stromal invasion was absent. Lymph node implants were defined as ovarian epithelial proliferations in the subcapsular sinuses, or single cells and small clusters of rounded cells with eosinophilic cytoplasm in the nodal sinuses, without the histological features of tissue invasion [40]. Peritoneal disease in ovarian tumors was referred to as implants and not metastases [41]. Implants were classified as non-invasive or invasive. Non-invasive implants were defined as desmoplastic or epithelial cells in peritoneal surfaces showing no invasion. Invasive implants were defined as extensive epithelial components, sometimes with glands, or small clusters of cells leading to destructive stromal invasion into underlying tissue [41]. Stromal microinvasion was defined as the presence of stromal invasion < 10 mm^2^. Staging of the disease was retrospectively performed according to the International Federation of Gynecology and Obstetrics (FIGO) staging system for ovarian carcinoma established in 2015. In cases of incomplete surgical staging, the stage was evaluated based on the patients’ operative records [42, 43], and on the available pathologic findings, with unevaluated areas considered negative for metastatic lesions.

The decision to perform adjuvant chemotherapy was generally based on the rupture of ovarian cysts, positive pelvic washing data, lymph node status, invasive implants, and/or the extent of disease. Cis/carboplatin and paclitaxel (PT) were the primary regimens and consisted of: PT (175–180 mg/m^2^) plus cisplatin (50–75 mg/m^2^) or carboplatin (AUC = 5) given intravenously on day 1, every 4 weeks × (3–4) cycles.

After the completion of the initial treatment, patients were followed up once a month for the first 6 months, every 3 months for the next 6 months, every 6 months for the next 2 to 5 years, and every year thereafter. Efforts were made to contact patients who did not attend regular follow-up appointments by phone call or letter to obtain the required information. Recurrence was defined by clinical and/or imaging evidence and was confirmed pathologically.

### Statistical analysis

All statistical analyses were performed using SPSS version 22.0 (SPSS Inc, Chicago, IL). The Kaplan-Meier method was used for a univariate analysis of disease-free survival (DFS) and overall survival (OS). Different survival curves were compared using the log-rank test. DFS was calculated in months from the date of initial surgery to the date of recurrence; patients who survived or died of other conditions at the time of their last visit were censored. OS was calculated in months from the date of the initial surgery to the date of the patient’s death; patients who were disease free at the time of their last visit were censored. The Cox proportional hazard model (Wald stepwise backward regression) was utilized to evaluate all parameters that were significant in univariate analyses. The multivariate adjusted odds ratios (ORs) and 95% confidence intervals (CIs) are expressed. All the tests were 2 sided, and *p*-values lower than 0.05 were considered statistically significant.

## Abbreviations

BOTs: Borderline ovarian tumors
DFS: Disease-free survival
OS: Overall Survival
FIGO: International Federation of Gynecology and Obstetrics
